# Predischarge Car Seat Tolerance Screening in Preterm and At-Risk Full-Term Infants: A Systematic Review and Meta-Analysis

**DOI:** 10.1001/jamanetworkopen.2025.58197

**Published:** 2026-02-09

**Authors:** Brian C. King, Nisha Dalvie, Susanne Hay, Erik A. Jensen, John A. F. Zupancic

**Affiliations:** 1 Department of Neonatology, Beth Israel Deaconess Medical Center, Boston, Massachusetts; 2 Division of Newborn Medicine, Harvard Medical School, Boston, Massachusetts; 3 Division of Newborn Medicine, Boston Children’s Hospital, Boston, Massachusetts; 4 Division of Neonatal and Developmental Medicine, Department of Pediatrics, Stanford University School of Medicine, Lucile Packard Children’s Hospital, Palo Alto, California

## Abstract

**Importance:**

Predischarge car seat tolerance screening (CSTS) has been recommended by the American Academy of Pediatrics since 1991 for preterm and at-risk full-term–born infants. However, it remains unclear whether routine CSTS prevents adverse outcomes after discharge.

**Objective:**

To estimate the frequency of failed CSTS and its association with adverse postdischarge outcomes.

**Data Sources:**

PubMed, Embase, and Web of Science were searched for English-language studies published before June 2025.

**Study Selection:**

Randomized trials, nonrandomized intervention studies (utilizing a comparison group discharged without CSTS), and single-group observational studies were eligible.

**Data Extraction and Synthesis:**

Data were extracted following the Preferred Reporting Items for Systematic Reviews and Meta-Analyses reporting guideline by 2 reviewers. Study quality was assessed using the Risk of Bias in Nonrandomized Studies tool.

**Main Outcomes and Measures:**

Outcomes were 30-day readmission, mortality, and predischarge length of stay for intervention studies, and first and subsequent CSTS failure rates for single-group studies. Random-effects models were used to pool data, and generalized linear mixed models were used to estimate pooled treatment effects from 2-group studies and CSTS failure event rates from all included studies.

**Results:**

A total of 21 studies were included. No randomized trials were identified. Three nonrandomized intervention studies (54 358 participants; 27 786 participants without CSTS) reported postdischarge outcomes. There was no difference in 30-day mortality (2 studies; not pooled due to 0 events), 30-day readmissions (odds ratio, 1.05; 95% CI, 0.86-1.28; 3 studies; 54 559 participants), or combined 30-day mortality or readmission (odds ratio, 1.17; 95% CI, 0.95-1.43; 2 studies; 49 420 participants) among infants receiving predischarge CSTS compared with those that did not. Pooled analysis estimated 8.62 (95% CI, 6.42-11.47) first-test failures per 100 patients (21 studies; 39 052 participants) and 24.40 (95% CI, 6.44-34.64) repeat-test failures per 100 patients (11 studies; 912 participants).

**Conclusions and Relevance:**

In this systematic review and meta-analysis of predischarge CSTS for preterm and at-risk full-term–born infants, CSTS was not associated with a reduction in postdischarge readmission or mortality. These findings call into question whether routine CSTS before discharge improves outcomes in preterm or at-risk full-term infants.

## Key Points

**Question:**

Is routine predischarge car seat tolerance screening (CSTS) associated with fewer adverse postdischarge outcomes in preterm and at-risk full-term–born infants?

**Findings:**

In this systematic review and meta-analysis of 21 studies, including 3 nonrandomized intervention studies with 54 358 participants, CSTS was not associated with reduced 30-day mortality or hospital readmission. Approximately 9% of infants failed the first test and 24% of those failed a repeat test 24 to 72 hours later.

**Meaning:**

These findings suggest that routine CSTS may prolong hospitalization when infants fail CSTS, without clear evidence of postdischarge benefit, suggesting the need to reevaluate its widespread use.

## Teaser

This systematic review and meta-analysis measures the frequency of failed car seat tolerance screening and associations with adverse postdischarge outcomes among preterm and at-risk full term–born infants.

## Introduction

A predischarge car seat tolerance screening (CSTS), in which a newborn is positioned in a car seat and undergoes continuous observation and cardiorespiratory monitoring for a fixed period of time prior to discharge, has become standard of care for preterm infants and at-risk full-term newborns in the US since the American Academy of Pediatrics first endorsed the test in 1991.^1^ That recommendation was based on observations from small cohort studies showing that preterm infants and full-term–born infants with risk factors (eg, low birth weight) can exhibit vital sign instability when semi-upright in a car seat.^2,3^ Subsequent studies have confirmed these observations,^4,5,6^ and the American Academy of Pediatrics has continued to reaffirm the recommendation. Adherence to the policy is generally high, with more than 90% of nurseries and neonatal intensive care units (NICUs) reporting routine use, but with substantial intercenter variability in eligibility criteria, thresholds that define CSTS failure, and retesting strategies.^7,8,9^

The goal of CSTS is to accurately identify infants who are at risk of adverse outcomes due to vital sign instability that may occur during travel in a car seat and is otherwise preventable by a delay in discharge or other intervention. While studies have continued to investigate variations in CSTS eligibility and failure criteria, few have focused on whether routine CSTS prevents harm or improves patient outcomes. A Cochrane systematic review published in 2006 found no randomized clinical trials that addressed this question.^10^ Nonetheless, there has been a shift away from routine CSTS in some centers and regions. The Canadian Paediatric Society removed recommendations for CSTS in 2016, citing a lack of supportive evidence.^11^ Southern California Kaiser Permanente also instituted a policy change discontinuing routine CSTS in 2016. Published data from that network, inclusive of more than 40 000 infants, showed no change in the rates of measured adverse outcomes after this policy change.^12^ Motivated by these reports, we undertook the present study to systematically review the literature to identify studies in which CSTS was evaluated as an intervention with comparison with a control group of infants who did not undergo testing and to estimate the association of CSTS vs nonscreening with postdischarge outcomes. As a secondary objective, we sought to identify all studies reporting CSTS failure rates to estimate the association of CSTS with discharge and length of stay among newborn infants.

## Methods

We conducted a systematic review and meta-analysis to estimate the association of CSTS with length of stay and postdischarge outcomes and to estimate the overall proportion of infants who fail an initial and subsequent test. Reporting follows the Preferred Reporting Items for Systematic Reviews and Meta-Analyses (PRISMA) reporting guideline.^13^ The review was prospectively registered on PROSPERO (CRD42025635384).

### Eligibility Criteria

Full-length English-language articles published before June 2025 were included if they met the following inclusion criteria: (1) newborn population prior to first hospital discharge and (2) CSTS with established failure criteria. All study types were considered and were categorized into 3 subgroups: (1) randomized clinical trials, (2) nonrandomized intervention studies that included a comparison group of infants that did not undergo or have a documented passed CSTS prior to discharge, and (3) single-group observational studies reporting CSTS failure rates. Exclusion criteria were: (1) observational studies that described clinical instability without reporting CSTS failure, (2) observational studies of adherence to testing recommendations, (3) surveys of clinical practice, and (4) studies that were limited to subgroups beyond gestational age and birth weight (eg, congenital heart disease).

### Search Strategy

The literature search, conducted in accordance with PRISMA guidelines,^13^ was performed in June 2025 using PubMed (MEDLINE), Embase, and Web of Science, supplemented by manual review of bibliographies from retrieved articles. Search strategies for each data source are included in eTable 1 in Supplement 1. Two authors (B.C.K. and N.D.) reviewed titles, abstracts, and full texts of identified articles, as necessary, to determine eligibility and perform extraction in duplicate.

### Data Extraction

Data extracted from all eligible studies included study design, number of infants, gestational age, birth weight ranges, CSTS eligibility, and failure criteria. Extracted in-hospital outcomes (if available) were rates of first and subsequent test failure, timing of repeat test, and length of stay. Postdischarge outcomes (if available) were death, hospital readmission, and neurodevelopmental impairment. Data for postdischarge outcomes were only extracted from studies that compared infants who underwent CSTS with those who did not or those discharged without a documented passed CSTS. Outcomes among key subgroups defined by (1) admission to the NICU vs newborn nursery at the time of CSTS and (2) gestational age (<37 weeks and full-term–born infants) were also extracted. In cases where insufficient data were reported for pooling of a specific outcome (eg, a missing denominator), the study was excluded from the pooled estimate for that outcome.

### Risk of Bias and Certainty of Evidence

Risk of bias among nonrandomized intervention studies was formally evaluated using the Risk of Bias in Nonrandomized Studies of Interventions (ROBINS-I v2) tool, and visualized using the RobVis online tool.^14,15^ Grading of Recommendations Assessment, Development, and Evaluation criteria were used to assess certainty of evidence for the associations of CSTS with predischarge length of stay and postdischarge outcomes.^16^

### Statistical Analysis

Outcomes were reported as odds ratios (ORs) with 95% CIs for dichotomous outcomes or proportions with 95% CIs for single-group studies. Effect size estimates for comparative studies (randomized trials and nonrandomized intervention studies) were calculated using generalized linear mixed models (GLMMs) with a binomial likelihood and a logit link. For each study, the model estimates the log-odds of failure in the CSTS group and the comparison group and provides a study-specific OR with a 95% CI derived directly from the GLMM. For observational studies without a comparison group, the proportion of infants failing the CSTS was calculated as the number of failures divided by the total number tested. Individual-study 95% CIs were computed using exact Clopper-Pearson binomial limits to ensure appropriate coverage for small samples and rare events. Pooled estimates were obtained using random-effects GLMMs for both 2-group (ORs) and single-group (proportions) meta-analyses. GLMM was chosen because it models event counts and sample sizes directly, incorporates study-level random effects, and provides appropriate estimation of between-study heterogeneity when event counts are low. In cases where outcomes were too heterogeneous to support pooling (as determined by study author consensus), results were described narratively. Heterogeneity was explored descriptively, particularly in relation to CSTS eligibility and failure criteria. All analyses were conducted in R version 4.5.1 (R Project for Statistical Computing) using the meta package,^17^ and illustrative code for the analysis is included in eTable 2 in Supplement 1.

## Results

**Figure 1.**
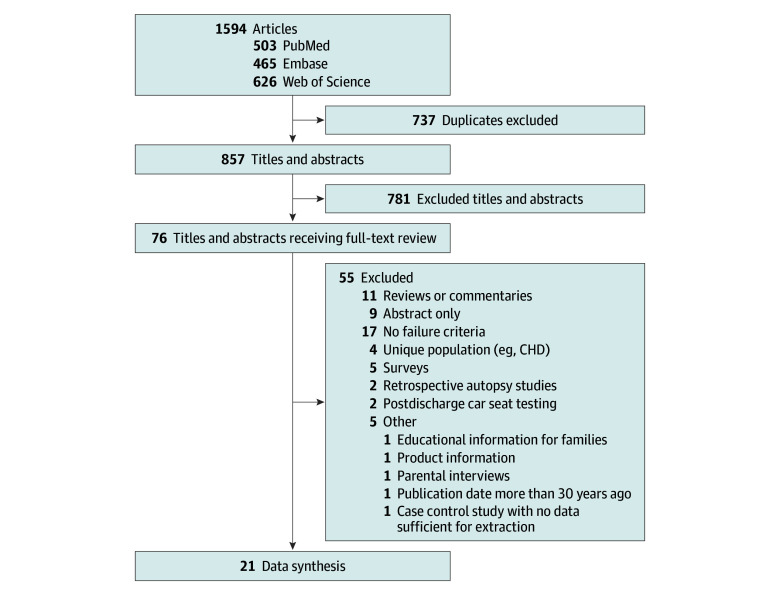
Study Flow Diagram CHD indicates congenital heart disease.

Systematic review of the literature yielded 857 unique articles. Title and abstract review identified 76 articles for full text review, of which 55 were excluded, leaving 21 studies^5,12,18,19,20,21,22,23,24,25,26,27,28,29,30,31,32,33,34,35,36^ for data synthesis (Figure 1).

### Description of Included Studies

No randomized clinical trials were identified. Most studies were single-group and lacked a comparison group of infants who did not undergo CSTS. Three studies^12,18,19^ (14%) included a comparison group discharged without a passed CSTS and were categorized as nonrandomized interventional studies (54 358 participants; 27 786 participants without CSTS). The first was a multicenter retrospective cohort study by Jensen et al^19^ in which all patients underwent CSTS, with outcomes reported for a group of patients without a documented passed CSTS prior to discharge. The second was a 2-center retrospective cohort study of late preterm and term low birth weight infants by Harrison et al,^18^ with a population of infants that met screening criteria but did not undergo CSTS. The final study, by Braun et al,^12^ compared outcomes in a multicenter hospital system before and after a policy change that stopped routine CSTS. Risk of bias assessments of the included nonrandomized intervention studies are summarized in eFigure 1 in Supplement 1.

Data on the reported population, location of testing, and failure criteria for the included studies are summarized in Table 1. Most studies were of preterm infants (gestational age <37 weeks; 14 studies^12,19,20,22,23,25,26,27,28,29,31,32,34,35^ [67%]) and included patients admitted to either the NICU or well-baby nurseries (15 studies^5,12,18,20,21,22,23,24,25,26,27,28,31,32,35^ [71%]); ten studies^5,18,20,21,22,23,24,25,28,35^ reported results separately for each location, which allowed for subgroup analyses. Regarding CSTS failure criteria, all included studies were consistent with their definition of apnea (20 seconds) but used variable thresholds to define bradycardia and desaturation events (Table 1). Individual descriptions of included studies are included in eTables 3 to 5 in Supplement 1.

**Table 1.** Study Characteristics and Car Seat Tolerance Screening Failure Criteria Among Included Studies

Characteristic	Studies, No. (%) (N = 21)
Study characteristics	
Gestational age criteria	
<37 wk	14 (67)^12,19,20,22,23,25,26,27,28,29,31,32,34,35^
<35 wk	1 (5)^33^
34-36 wk	2 (9)^18,21^
34-37 wk	1 (5)^24^
30-37 wk	1 (5)^28^
>37 wk	2 (9)^5,30^
Birth weight criteria	
<2500 g	3 (14)^5,27,36^
<2270 g	3 (14)^18,22,23^
<1500 g	1 (5)^34^
Small for gestational age	
Reported	1 (5)^35^
None or not reported	13 (62)^12,19,20,21,24,25,26,28,29,30,31,32,33^
Location	
Nursery only	2 (10)^30,36^
Neonatal intensive care unit only	4 (19)^19,28,33,34^
Both	15 (71)^5,12,18,20,21,22,23,24,25,26,27,29,31,32,35^
Study design	
Randomized clinical trial	0
Nonrandomized intervention study	3 (14)^12,18,19^
Single-group observational study	18 (86)^5,20,21,22,23,24,25,26,27,28,29,30,31,32,33,34,35,36^
Test failure criteria	
Apnea duration	
20 s	16 (76)^5,18,20,21,22,25,26,27,28,29,30,31,33,34,35,36^
Not described	5 (24)^12,19,23,24,32^
Bradycardia cutoff	
<80 bpm	16 (76)^5,20,21,22,24,25,26,27,28,29,30,32,33,34,35,36^
Combination	1 (5)^18^
Not described	4 (19)^12,19,23,31^
Bradycardia duration, s	
Any	3 (14)^5,27,33^
10	6 (28)^18,20,21,22,29,36^
5	2 (10)^34,35^
Persistent	2 (10)^26,30^
Combination	1 (5)^32^
Not described	7 (33)^12,19,23,24,25,28,31^
Desaturation cutoff, % saturation	
93	2 (9)^25,29^
92	1 (4)^22^
90	5 (24)^21,26,28,30,31^
88	4 (19)^5,20,27,34^
85	2 (10)^35,36^
Combination	4 (19)^18,24,32,33^
Not described	3 (14)^12,13,16^
Desaturation duration, s	
Any	2 (9)^5,27^
20	1 (5)^20^
10	6 (29)^21,22,28,29,34,36^
5	1 (5)^35^
Persistent or recurrent	3 (14)^26,30,31^
Combination	4 (19)^18,24,32,33^
Not described	4 (19)^12,19,23,25^

### First Test Failure

**Figure 2.**
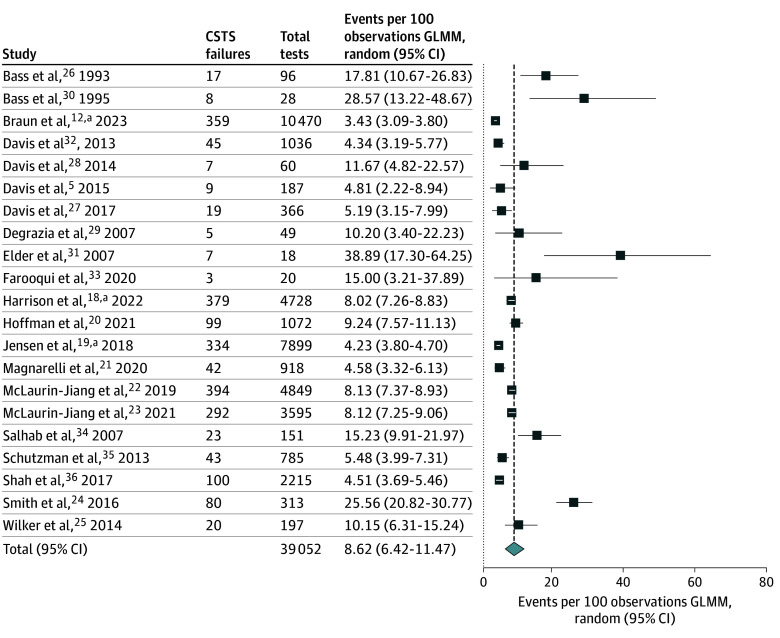
Single-Group Meta-Analysis of All Included Studies Reporting First Test Failure Rate for Car Seat Tolerance Screening (CSTS) Before Discharge GLMM indicates generalized linear mixed models. ^a^Meta-analysis incorporates CSTS results from patients who underwent CSTS in nonrandomized intervention studies that included a comparison group.

Among the 21 included studies there was an overall estimate of 8.62 (95% CI, 6.42-11.47) CSTS failures per 100 patients (39 052 participants) (Figure 2). Among the subgroup of patients undergoing testing in the nursery, the estimate was 9.86 (95% CI, 6.75-14.18) failures per 100 patients (12 021 participants; 10 studies^5,18,20,21,22,23,24,25,30,36^) compared with 7.11 (95% CI, 5.49-9.17) failures per 100 patients (14 121 participants; 13 studies^5,18,19,20,21,22,23,24,25,28,33,34,35^) among infants in the NICU (eFigure 2 in Supplement 1). Eight studies^5,18,20,21,22,23,24,25^ included patients undergoing initial testing in the nursery or NICU, and in 6 of those studies^5,18,20,21,22,24^ the first test failure rate was higher in the nursery. Among preterm infants, the overall estimate was 8.56 (95% CI, 6.29-11.55) failures per 100 patients (34 685 participants; 18 studies^12,18,19,20,21,22,23,24,25,26,27,28,29,31,32,33,34,35^), compared with an estimate of 10.72 (95% CI, 7.27-15.51) failures per 100 patients among full-term–born infants (1966 participants; 8 studies^5,18,20,22,25,26,27,30^) (eFigure 3 in Supplement 1). Six studies^18,20,22,25,26,27^ included both preterm and full-term–born infants undergoing their initial test, with 5 studies^18,20,22,25,27^ reporting higher first test failure rates among term infants.

### Repeat CSTS

**Figure 3.**
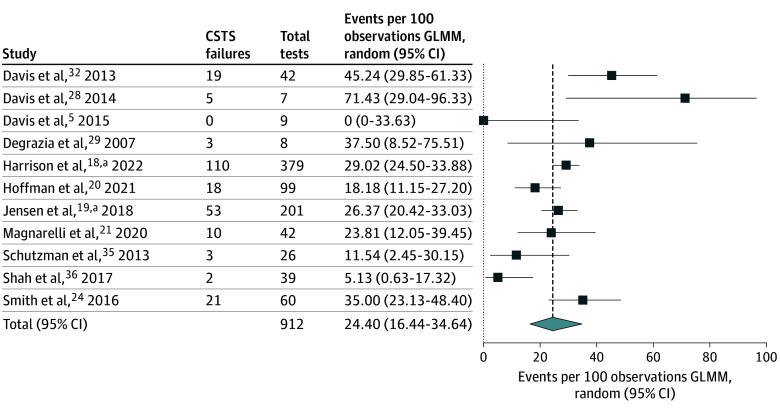
Single-Group Meta-Analysis of All Included Studies Reporting Repeat Test Failure Rate for Car Seat Tolerance Screening (CSTS) Before Discharge GLMM indicates generalized linear mixed models. ^a^Meta-analysis incorporates CSTS results from patients who underwent CSTS in nonrandomized intervention studies that included a comparison group.

Eleven studies^5,18,19,20,21,24,28,29,32,35,36^ with a total of 912 participants reported results for repeat CSTS among infants who failed initial screening. The timing of repeat testing was variable. In 6 studies,^5,20,24,28,29,36^ repeat testing was performed between 12 and 48 hours after the initial test, in 2 studies^19,32^ repeat testing was delayed for more than 48 hours after initial testing, and 3 studies^18,21,24^ did not report the interval. The overall estimate for the 11 studies was 24.40 (95% CI, 16.44-34.64) repeat CSTS failures per 100 patients (Figure 3). Data were not sufficient for pooling repeat testing results among subgroups of infants by gestational age. Two studies^28,29^ reported results of repeat testing among infants regardless of whether they failed their initial test (test-retest reliability). In Davis et al,^28^ 11% of moderately preterm infants (6 of 53 infants) who passed an initial car seat test failed 1 of 2 repeats 24 to 48 hours later. In DeGrazia et al,^29^ among the 41 preterm infants who passed their initial car seat test, 4 (10%) failed repeat testing 12 to 36 hours later.

### Predischarge Length of Stay

Only 2 studies^12,18^ compared predischarge length of stay between patients who underwent CSTS before discharge and patients discharged without CSTS. Due to differences in study population, study design, and outcome reporting, data from these studies were not combined in a pooled analysis. Braun et al^12^ reported no difference in unadjusted overall length of stay (14.24 vs 14.26 days; *P* = .96) when comparing 21 122 infants before and 20 142 infants after discontinuation of routine CSTS. Harrison et al^18^ reported no significant difference in length of stay (adjusted length of stay, 6.1 hours longer [95% CI, 1.2 hours shorter to 13.3 hours longer] for screened patients). However, in a stratified analysis, Harrison et al^18^ found that use of routine CSTS was associated with a significantly longer adjusted length of stay (35.5 hours longer [95% CI, 13.5 to 57.5 hours longer]) among infants hospitalized in the NICU. Based on these 2 studies,^12,18^ there is very low certainty of evidence that car seat testing is associated with overall average predischarge length of stay.

### Postdischarge Outcomes

Three studies^12,18,19^ described postdischarge outcomes with a comparison group of infants who either did not undergo CSTS or did not have a documented passed test before discharge. Due to differences in study design and reporting, outcomes from these studies are primarily presented separately (Table 2). Two studies^12,19^ reported death within 30 days of discharge, but Jensen et al^19^ observed no deaths in the cohort of infants discharged without a passed CSTS. As a result, data from the 2 studies were not pooled in a meta-analysis. Braun et al^12^ observed fewer deaths after stopping routine predischarge CSTS (OR, 0.26; 95% CI, 0.11-0.64); however, after adjustment for other clinical factors, the difference was not significant (adjusted OR, 0.94; 95% CI, 0.72-1.22). All 3 studies reported no significant differences in rates of readmission within 30 days after discharge. Meta-analysis of data from the 3 studies similarly showed no difference in unadjusted readmission rate (OR. 1.05; 95% CI, 0.86-1.28; random-effects model; 54 559 participants; very low certainty) (eFigure 4 in the Supplement 1). Two studies^12,19^ included death or readmission within 30 days as an outcome, and there were no significant differences in the composite outcome between the groups in either study, nor in the pooled rate of death or readmission within 30 days (OR, 1.17; 95% CI, 0.95-1.43; random-effects model; 49 420 participants; very low certainty) (eFigure 4 in the Supplement 1). None of the included studies reported on neurodevelopmental outcomes.

**Table 2.** 30-Day Postdischarge Outcomes Among Included Nonrandomized Intervention Studies

30-d Postdischarge outcome	Participants, No. (%)		OR (95% CI)	*P* value	Adjusted OR (95% CI)^b^	Adjusted *P* value
	CSTS	No CSTS^a^				
Death						
Braun et al,^12^ 2023	24 (0.12)	6 (0.02)	0.26 (0.11-0.64)	.004	0.94 (0.72-1.22)	.63
Jensen et al,^19^ 2018	3 (0.04)	0	NA^c^	>.99	NA^c^	NA
Readmission^d^						
Braun et al,^12^ 2023						
Brief resolved unexplained events	133 (0.62)	108 (0.54)	0.93 (0.71-1.23)	.61	0.69 (0.45-1.04)	.08
Respiratory diagnoses	46 (0.22)	49 (0.24)	1.33 (0.87-2.06)	.19	1.18 (0.65-2.13)	.59
Jensen et al,^19^ 2018	80 (1.00)	3 (2.00)	0.51 (0.16-1.64)^e^	.21	1.20 (0.14-10.2)	.87^e^
Harrison et al,^18^ 2022	137 (2.90)	15 (7.30)	0.69 (0.40-1.20)	.19^e^	0.78 (0.44-1.40)	.40^e^
Death or readmission^f^						
Braun et al,^12^ 2023	217 (1.02)	213 (1.06)	1.19 (0.85-1.67)	.30	0.94 (0.72-1.22)	.63
Jensen et al,^19^ 2018	83 (1.10)	3 (2.00)	0.53 (0.17-1.70)^e^	.22	1.25 (0.16-9.82)	.83^3^

Abbreviations: CSTS, car seat tolerance screening; NA, not applicable; OR, odds ratio.

^a^ The comparison group in Braun et al^12^ and Harrison et al^18^ were infants who did not undergo any CSTS. The comparison group in Jensen et al^19^ was a population of infants that failed an initial CSTS and were discharged home without a passed CSTS.

^b^ All 3 studies^12,18,19^ used multivariable logistic regression to estimate adjusted ORs, including both maternal and infant characteristics as well as hospital of birth. All 3 outcomes were rated as very low certainty of evidence, due to the observational study design, risk of bias, inconsistency, and imprecision.

^c^ No calculated OR or adjusted OR provided due to the lack of events in the no CSTS group.

^d^ A combined readmission outcome for the 2 subgroups is not reported.

^e^ OR or *P* values were not reported in the original study but were estimated using the Wald method from data provided.

^f^ For Braun et al,^12^ the composite of death or readmission also includes 911 transport calls, and data were not provided to exclude calls that did not result in a hospital readmission.

## Discussion

In this systematic review and meta-analysis, we identified no randomized trials of CSTS, and only 3 nonrandomized intervention studies of CSTS reporting postdischarge outcomes. The overall pooled estimate suggested approximately 9% (95% CI, 8%-12%) of infants fail prior to discharge, with a further 25% failing repeat testing, typically at least 1 day later, albeit with wide variation in reported CSTS eligibility and failure criteria. There was no significant difference in predischarge length of stay or postdischarge outcomes (all very low certainty evidence) associated with routine CSTS vs nontesting or discharge following failed CSTS among the 3 identified studies.

Although the average length of stay was not different between screened and unscreened populations, test-retest characteristics allow a conservative estimate of the potential association of CSTS with hospitalization. If the approximately 300 000 infants who are born preterm in the US each year undergo testing and approximately 10% fail their first CSTS, more than 30 000 infants annually may have discharge delayed by at least 12 to 24 hours. If approximately 25% of those who undergo repeat screening fail subsequent testing, we estimate that routine CSTS could contribute to more than 40 000 additional hospital days and an estimated $25 to $50 million in additional annual direct medical costs.^37^ This estimate is likely conservative because it does not consider additional test failures or full-term–born infants who selectively undergo testing based on risk factors. Formal economic evaluation would allow for more precise estimation of the financial impact of CSTS failures.

Delaying hospital discharge due to CSTS would be beneficial, provided the test and subsequent delay prevents adverse outcomes. A prior Cochrane systematic review^10^ highlighted the lack of evidence that CSTS improves outcomes following discharge, but importantly only sought evidence from randomized trials. This updated systematic review, nearly 20 years later, similarly identified no published trials of CSTS. In addition, we specifically sought to include studies using other methodologies to address this question and identified 3 eligible studies,^12,18,19^ all of which reported no association of predischarge CSTS with postdischarge mortality or readmission. Importantly, no studies have reported neurodevelopmental outcomes among infants who do or do not undergo CSTS. The long-term neurodevelopmental significance of cardiopulmonary events that occur during car travel and may be impacted in some by routine predischarge CSTS is therefore unknown, but may be of questionable importance compared with the relatively high frequency of events known to occur at home in healthy full-term–born infants, regardless of positioning, and the overall low expected daily duration of travel in an infant car seat.^38^

Two other aspects of the CSTS highlighted by this review further add to the concern that the test may not be an appropriate screening tool to identify infants at risk for postdischarge adverse outcomes. The first is the high observed rate that infants pass repeat testing after a short interval (75% will pass a subsequent test in 12 to 48 hours). Such rapid improvement is unlikely to reflect true physiologic maturation, raising questions about whether CSTS meaningfully identifies at-risk infants. In addition, 2 studies^28,29^ reported retest reliability among infants with passed initial screening and in both approximately 10% failed a retest. Assuming these events represent false positives and false negatives, the sensitivity and specificity of the initial CSTS result would be 90% and 25%, respectively. Moreover, it remains uncertain whether even a subsequent test failure is a risk factor for adverse events following discharge.

The initial endorsement by the American Academy of Pediatrics, and subsequent reaffirmations, have led to relatively high adherence to predischarge CSTS, despite the lack of evidence of benefit.^7,8^ As a result, there is still hesitancy toward formal evaluation of the intervention. In a survey of attendees at an international neonatology conference, Jensen et al^39^ found that 60% of respondents lacked equipoise for a hypothetical trial that would randomize one-half of homegoing preterm infants to discharge without routine CSTS. This lack of personal equipoise is in contrast with the clinical equipoise that exists, as evidenced by recent policy changes by Kaiser Permanente Southern California and the Canadian Paediatric Society.^11,12^ This systematic review also shows uncertainty in benefit with routine CSTS, which support equipoise to conduct a trial, if it could be feasibly designed to reliably answer whether screening is effective.

### Strengths and Limitations

Strengths of this review include a structured approach to the literature search and extraction of data, and the inclusion of nonrandomized studies. This approach allowed us to summarize evidence from multiple study types, while still formally assessing the risk of bias and certainty of evidence.

Limitations from this study include the heterogeneity in testing eligibility and failure criteria among included studies, which contributed to wide confidence intervals. We attempted to address this in subgroup analyses but were limited by the data included in published reports. Three studies in particular were noted to have higher failure rates than other included studies, which could not be explained in subgroup analysis by gestational age or location of testing.^24,30,31^ Two of those studies^30,31^ used small sample sizes, and the third (Smith et al^24^) only included moderately preterm infants, which may have unique risks for CSTS failure. There was also greater heterogeneity among studies reporting repeat test failures, which may be partly due to smaller sample sizes among included studies. Only 3 nonrandomized studies^12,18,19^ reported postdischarge outcomes, with 1 study^3^ contributing the majority of participants. As a result, pooled estimates for these rare events were imprecise, model-dependent, and did not permit robust assessment of heterogeneity or publication bias, yielding a very low certainty of evidence. Nonetheless, because CSTS is already embedded in routine practice, synthesizing the best available evidence remains important to inform current policy and clinical decision-making. It is also important to acknowledge that even if recommendations regarding routine CSTS merit reevaluation, these findings do not diminish the importance of appropriate car seat selection, fit, and proper use, which are essential for safe transport of all infants and young children.

## Conclusions

This systematic review and meta-analysis identified no published randomized trials of CSTS; across 3 nonrandomized studies, CSTS was not associated with reductions in 30-day mortality or hospital readmission (very low certainty evidence). Approximately 9% of infants fail initial testing, of whom 1 in 4 fail repeat CSTS. These failure events potentially contribute to prolonged hospitalization in this subset of infants without clear evidence of benefit. As such, current recommendations for routine CSTS in all preterm infants may merit reevaluation.

## Notes

Supplement 1. eTable 1. Search strategies for included databases eTable 2. Illustrative code used for meta-analysis eTable 3. Description of included studies eTable 4. Complete data extraction for CSTS test results eTable 5. Complete data extraction for clinical outcomes among non-randomized intervention studies eFigure 1. Risk of bias assessment for included non-randomized intervention studies using the ROBINS-I v2 tool eFigure 2. Subgroup analysis of single-arm meta-analysis of first test CSTS failure among infants admitted to the A) Nursery and B) NICU eFigure 3. Subgroup analysis of single-arm meta-analysis of first test CSTS failure among A) Preterm infants and B) Term infants eFigure 4. Post-discharge outcomes from included studies comparing CSTS vs no CSTS

Supplement 2. Data Sharing Statement
