# Supplementary material for: Predischarge Car Seat Tolerance Screening in Preterm and At-Risk Full-Term Infants: A Systematic Review and Meta-Analysis
Source: JAMA Netw Open. 2026 Feb 9;9(2):e2558197. doi: 10.1001/jamanetworkopen.2025.58197 (PMC12887743; doi:10.1001/jamanetworkopen.2025.58197)
Supplement: Supplement 1. — eTable 1. Search strategies for included databases eTable 2. Illustrative code used for meta-analysis eTable 3. Description of included studies eTable 4. Complete data extraction for CSTS test results eTable 5. Complete data extraction for clinical outcomes among non-randomized intervention studies eFigure 1. Risk of bias assessment for included non-randomized intervention studies using the ROBINS-I v2 tool eFigure 2. Subgroup analysis of single-arm meta-analysis of first test CSTS failure among infants admitted to the A) Nursery and B) NICU eFigure 3. Subgroup analysis of single-arm meta-analysis of first test CSTS failure among A) Preterm infants and B) Term infants eFigure 4. Post-discharge outcomes from included studies comparing CSTS vs no CSTS [file jamanetwopen-e2558197-s001.pdf]

Supplemental Online Content

King BC, Dalvie N, Hay S, Jensen EA, Zupancic JAF. Predischarge car seat tolerance screening in preterm and at-risk full-term infants: a systematic review and meta-analysis. *JAMA Netw. Open*. 2026;9(2):e2558197.
doi:10.1001/jamanetworkopen.2025.58197

eTable 1. Search strategies for included databases

eTable 2. Illustrative code used for meta-analysis

eFigure 1. Risk of bias assessment for included non-randomized intervention studies using the ROBINS-I v2 tool

eTable 3. Description of included studies

eTable 4. Complete data extraction for CSTS test results

eTable 5. Complete data extraction for clinical outcomes among non-randomized intervention studies

eFigure 2. Subgroup analysis of single-arm meta-analysis of first test CSTS failure among infants admitted to the A) Nursery and B) NICU

eFigure 3. Subgroup analysis of single-arm meta-analysis of first test CSTS failure among A) Preterm infants and B) Term infants

eFigure 4. Post-discharge outcomes from included studies comparing CSTS vs no CSTS

This supplemental material has been provided by the authors to give readers additional information about their work.

© 2026 King BC et al. *JAMA Network Open*.

eTable 1. Search strategy for included databases.

Database	Search Strategy
Pubmed	("car seat*" OR "car safety seat") AND ("challenge*" OR "tolerance*" OR "test*" OR "safety*" OR "screen*" OR "hypoxia*" OR "hypoxemia*")
EMBASE	('car seat'/exp OR 'car seat' OR 'car safety seat') AND ('challenge*' OR 'tolerance*' OR 'test*' OR 'safety*' OR 'screen*' OR 'hypoxia*' OR 'hypoxemia*')
Web of Science	(ALL="car seat*" OR ALL="car safety seat") AND (ALL="challenge*" OR ALL="tolerance*" OR ALL="test*" OR ALL="safety*" OR ALL="screen*" OR ALL="hypoxia*" OR ALL="hypoxemia*")

© 2026 King BC et al. *JAMA Network Open*.

eTable 2. Illustrative code used for meta-analysis

<table>
<tr><th>Type of analysis</th><th>Illustrative code (meta package)</th></tr>
<tr><td>Single-arm meta-analysis (pooled proportions)</td><td>library(meta)
df <- read.csv("your_data.csv", stringsAsFactors=FALSE); names(df) <- trimws(names(df))

m_meta <- metaprop(event=Event, n=n, studlab=Author, data=df, method="GLMM", sm="PLOGIT")

study_labs <- with(df, mapply(function(a,r) if(is.na(r)||r=="") bquote(.(a)) else bquote(.(a)^.(r)), Author, Ref, SIMPLIFY=FALSE))

png("output.png", width=8, height=6, units="in", res=300); par(mar=c(10,4,4,8))
forest(m_meta, layout="Revman5", sortvar=studlab, x.lab="First test failure rate of first CSTS – All studies",
pscale=100, random=TRUE, common=FALSE, backtransf=TRUE, xlim=c(0,100), cex=0.7, fontsize=8,
studlab=study_labs, leftcols=c("studlab","event","n","effect.ci"),
leftlabs=c("Study","CSTS Failures","Total Tests",NA), colgap.forest="3cm")
dev.off()

summary(m_meta)</td></tr>
<tr><td>Non-randomized intervention studies</td><td>library(meta); df <- read.csv("death_or_readmit.csv")

outcome <- metabin(event.e=Event.CST, n.e=Event.CST+No.Event.CST,
event.c=Event.No.CST, n.c=Event.No.CST+No.Event.No.CST,
studlab=Author, data=df, sm="OR", method="GLMM",
random=TRUE, common=FALSE, label.e="CSTS", label.c="No CSTS",
label.left="Favours CSTS", label.right="Favours No CSTS")

study_labs <- with(df, mapply(function(a,r) if(is.na(r)||r=="") bquote(.(a)) else bquote(.(a)^.(r)),
Author, Ref, SIMPLIFY=FALSE))

png("death_or_readmit_GLMM.png", width=3000, height=1800, res=300); par(mar=c(7,10,4,8))
forest(outcome, studlab=study_labs); dev.off()</td></tr>
</table>

© 2026 King BC et al. *JAMA Network Open*.

eFigure 1. Risk of bias for included non-randomized intervention studies using the ROBINS-I v2 tool.

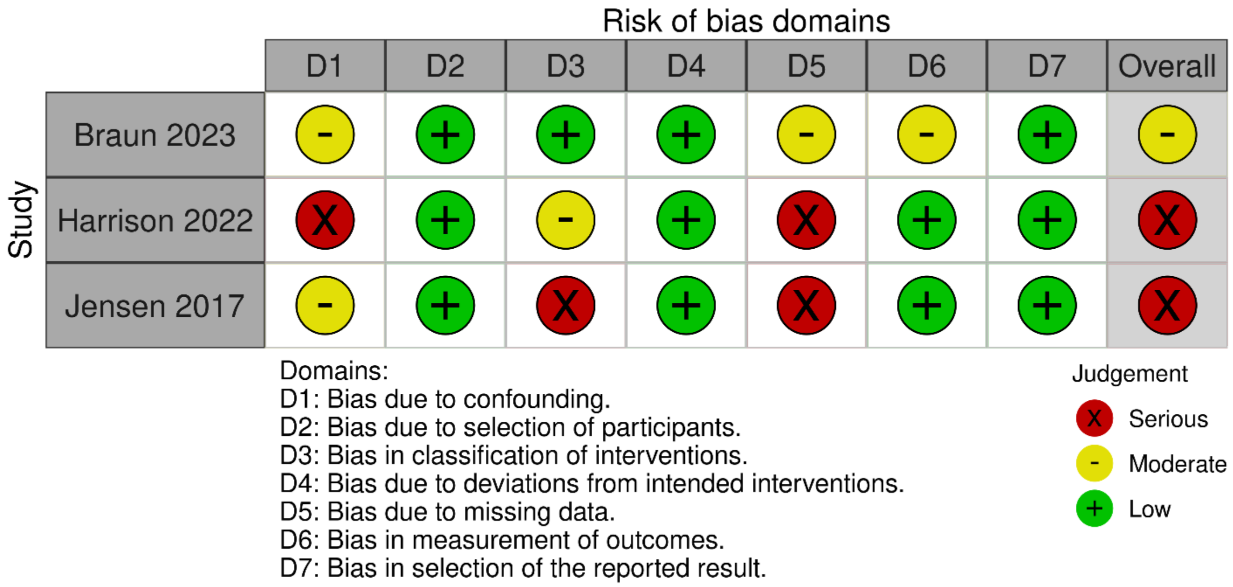

© 2026 King BC et al. *JAMA Network Open*.

eTable 3. Description of included studies.

Author Year	Population	Study Location	Study Design	Car seat test failure criteria	Timing of repeat car seat test
Bass et al,[26] 1993	GA <37 weeks<br>N = 87	NICU and Nursery	Prospective, single center, cohort study	Apnea – 20 seconds<br>Brady – 80 BPM, persistent<br>Desat – 90%, persistent	Not reported
Bass et al,[30] 1995	GA >37 weeks with risk factors<br>N = 28	Nursery only	Retrospective, single center, cohort study	Apnea – 20 seconds<br>Brady – 80 BPM, persistent<br>Desat – 90%, persistent	Not reported
Braun et al,[12] 2023	GA <37 weeks<br>N = 41,264 (n = 21,122 for CSTS period, n = 20,142 for CSTS discontinuation period)	NICU and Nursery	Retrospective, multicenter, pre/post observational cohort study	Not described	Not reported
Davis et al,[32] 2013	GA <37 weeks<br>N = 1,036	NICU and Nursery	Retrospective, multicenter, cohort study	Apnea – Not described<br>Brady – 80 BPM, duration varied by site (Any or 10 seconds)<br>Desat – 4 sites 88%, 2 sites 90%, duration varied by site (10-20 seconds)	Mean 7 days
Davis et al,[28] 2014	GA 30 to 37 weeks<br>N = 60	NICU only	Prospective, single center, cohort study (test-retest reliability)	Apnea – 20 seconds<br>Brady – 80 BPM, duration not described<br>Desat – 90%, 10 seconds	24 hours later
Davis et al,[5] 2015	GA >37 weeks and LBW	NICU and Nursery	Retrospective, single center, cohort study	Apnea – 20 seconds<br>Brady – 80 BPM, any duration<br>Desat – 88%, any duration	78% repeated 24 hours later
Davis et al,[27] 2017	GA <37 weeks or BW <2.5 kg<br>N = 366	NICU and Nursery	Retrospective, single center, cohort study	Apnea – 20 seconds<br>Brady – 80 BPM, any duration<br>Desat – 88%, any duration	Not reported
Degrazia et al,[29] 2007	GA <37 weeks<br>N = 49	NICU and Nursery	Prospective, single center, cohort study	Apnea – 20 seconds<br>Brady – 80 BPM, 10 seconds<br>Desat – 93%, 10 seconds	12 to 36 hours later
Elder et al,[31] 2007[9]	GA 24 to 36 weeks<br>N = 20	NICU and Nursery	Prospective, single center, cohort study	Apnea – 20 seconds<br>Brady – Not described<br>Desat – 90%, “recurrent”	Not reported
Farooqui et al,[33] 2020	GA <35 weeks<br>N = 20	NICU only	Prospective, single center, cohort study	Apnea – 20 seconds<br>Brady – 80 BPM, any duration<br>Desat – 80 or 85%, 10 or 20 seconds (respectively)	Not reported
Harrison et al,[18] 2022	GA 34-36 weeks or BW <2268 grams<br>N = 5,222 (n = 4,728 with CSTS, n = 494 without CSTS)	NICU and Nursery	Retrospective, multi-center, cohort study	Apnea – 20 seconds<br>Brady – Two sites (Site 1: 80 BPM, Site 2: 90 BPM, both 10 seconds)<br>Desat – Two sites (Site 1: 90%, 10 seconds, Site 2: 92%, 20 seconds)	Not reported
Hoffman et al,[20] 2021	GA <37 weeks or congenital anomalies<br>N = 1072	NICU and Nursery	Retrospective, single center, cohort study	Apnea – 20 seconds<br>Brady – 80 BPM, 10 seconds<br>Desat – 88%, 20 seconds	12 to 48 hours later

© 2026 King BC et al. *JAMA Network Open*.

Jensen et al,[19] 2018	GA <37 weeks<br>N = 7,899<br>(n = 7,749 with CSTS, n = 150 without CSTS)	NICU only	Retrospective, multi-center, cohort study	Not described	Average 2.1 days for those who failed subsequent test<br>Average 3.6 days for those who passed subsequent test
Magnarelli et al,[21] 2020	GA 34 to 36 weeks<br>N = 918	NICU and Nursery	Retrospective, single center cohort study	Apnea – 20 seconds<br>Brady – 80 BPM, 10 seconds<br>Desat – 90%, 10 seconds	Not reported
McLaurin-Jiang et al,[22] 2019	GA <37 weeks or BW < 2.27 kg<br>N = 4,849	NICU and Nursery	Retrospective, multi-center, cohort study	Apnea – 20 seconds<br>Brady – 80 BPM, 10 seconds<br>Desat – 92%, 10 seconds	Not reported
McLaurin-Jiang et al,[23] 2021	GA <37 weeks or BW <2.27 kg<br>N = 3,931	NICU and Nursery	Retrospective, multi-center, cohort study	Not described	Not reported
Salhab et al,[34] 2007	GA <37 weeks and BW <1.5 kg<br>N = 151	NICU only	Prospective, single center, randomized (crossover) study (car seat vs car bed)	Apnea – 20 seconds<br>Brady – 80 BPM, 5 seconds<br>Desat – 88%, 10 seconds	Not reported
Schutzman et al,[35] 2013	GA <37 weeks or SGA<br>N = 785	NICU and Nursery	Retrospective, single center, cohort study	Apnea – 20 seconds<br>Brady – 80 BPM, 5 seconds<br>Desat – 85%, 5 seconds	Not reported
Shah et al,[36] 2017	GA <37 weeks or BW <2.5 kg<br>N = 148 (failures only)	Nursery only	Retrospective, single center, cohort study	Apnea – 20 seconds<br>Brady – 80 BPM, 10 seconds<br>Desat – 85%, 10 seconds	24 to 48 hours later
Smith et al,[24] 2016	GA 34 to 37 weeks<br>N = 313	NICU and Nursery	Retrospective, single center, cohort study	Apnea – Not described<br>Brady – 80 BPM, duration not described<br>Desat – 80-88%, recurrent of 10 seconds (for 88%, once for 80%)	24 to 48 hours later
Wilker et al,[25] 2014	GA <37 weeks or congenital anomalies<br>N = 197	NICU and Nursery	Retrospective, single center, cohort study	Apnea – 20 seconds<br>Brady – 80 BPM, duration not described<br>Desat – 93%, duration not described	Not reported

© 2026 King BC et al. *JAMA Network Open*.

eTable 4. Complete data extraction for CSTS test results

Author Year	First CSTS (Full cohort)		First CSTS (NICU only)		First CSTS (Nursery only)		First CSTS (Preterm only)		First CSTS (Term only)		Repeat CSTS	
	**Test failure**	**Total tests**	**Test failure**	**Total tests**	**Test failure**	**Total tests**	**Test failure**	**Total tests**	**Test failure**	**Total tests**	**Test failure**	**Total tests**
Bass et al,[26] 1993	17	96	Not specified	Not specified	Not specified	Not specified	16	87	1	9	Not reported	Not reported
Bass et al,[30] 1995	8	28	N/A	N/A	8	28	N/A	N/A	8	28	Not reported	Not reported
Braun et al,[12] 2023[3]	359	10,470	Not specified	Not specified	Not specified	Not specified	359	10,470	Not applicable	Not applicable	Not reported	Not reported
Davis et al,[32] 2013	45	1036	17	No denominator[a]	28	No denominator[a]	45	1036	Not applicable	Not applicable	19	42
Davis et al,[28] 2014	7	60	7	60	N/A	N/A	7	60	N/A	N/A	5	7
Davis et al,[5] 2015	9	187	2	77	7	103	N/A	N/A	9	187	2	9
Davis et al,[27] 2017	19	366	Not specified	Not specified	Not specified	Not specified	13	293	6	73	Not reported	Not reported
Degrazia et al,[29] 2007	5	49	Not specified	Not specified	Not specified	Not specified	5	49	N/A	N/A	3	8
Elder et al,[31] 2007	7	18	Not specified	Not specified	Not specified	Not specified	7	18	N/A	N/A	Not reported	Not reported
Farooqui et al,[33] 2020	3	20	3	20	N/A	N/A	3	20	N/A	N/A	Not reported	Not reported
Harrison et al,[18] 2022	379	4728	80	1777	299	2951	340	4306	39	242	110	379
Hoffman et al,[20] 2021	99	1072	42	517	26	241	70	798	29	274	18	99
Jensen et al,[19] 2018	334	7899	334	7899	N/A	N/A	334	7899	N/A	N/A	53	201
Magnarelli et al,[21] 2020	42	918	26	497	16	339	42	918	N/A	N/A	10	42
McLaurin-Jiang et al,[22] 2019	394	4849	56	992	338	3857	299	3701	95	1148	Not reported	Not reported
McLaurin-Jiang et al,[23] 2021	292	3595	102	1679	190	1916	292	3595	N/A	N/A	Not reported	Not reported
Salhab et al,[34] 2007	23	151	23	151	N/A	N/A	23	151	N/A	N/A	Not reported	Not reported
Schutzman et al,[35] 2013	43	785	26	313	Not specified	Not specified	43	785	N/A	N/A	3	43
Shah et al,[36] 2017	100	2215	N/A	N/A	100	2215	102	No denominator[b]	46	No denominator[b]	2	39
Smith et al,[24] 2016	80	313	7	48	73	265	80	313	N/A	N/A	21	60
Wilker et al,[25] 2014	20	197	11	91	9	106	17	186	1	5	Not reported	Not reported

[a]Davis *et al.* 2013 report the total number of CSTS failures among a population of 1,036 infants. They report the proportion of these failures that occurred in the NICU and the nursery, but do not report the proportion of tests occurring in either setting, so this data was not included in pooled estimates.

[b]Shah *et al.* 2017 report 100 CSTS failures among 2215 tests, but also report 102 preterm CSTS failures and 46 term CSTS failures, without clearly specifying the discrepancy in the total number of failed tests, or providing a denominator of preterm and term infants who underwent CSTS. As a result, only the total number reported with a denominator was included in pooled estimates.

© 2026 King BC et al. *JAMA Network Open*.

eTable 5. Complete data extraction for clinical outcomes among non-randomized intervention studies.

	**Hospital length of stay**			**Post-discharge Readmission**	**Post-discharge Death**	**Other**
	**CSTS**	**No CSTS**	**Test of significance**			
Braun et al,[12] 2023	14.24 days (23.4), mean (SD)	14.26 days (25.3), mean (SD)	p = 0.96	See Table 2	See Table 2	911 call-triggered transports – 100 (0.48) vs 103 (0.52), p = 0.58 OR 1.08 (95% CI 0.82 to 1.42)
Harrison et al,[18] 2022	CSTS Pass – 87 hrs (62-185), median (IQR) CSTS Fail – 87 (69-137), median (IQR)	102 hrs (59-209), median (IQR)	Unadjusted OR -15.3 hrs (95% CI -22.6 to -8.1) Adjusted OR 6.1 (95% CI -1.2 to 13.3) Ref: Not screened	See Table 2	Not reported	N/A
Jensen et al,[19] 2018	Not reported	Not reported	Not reported	See Table 2	See Table 2	N/A

© 2026 King BC et al. *JAMA Network Open*.

eFigure 2. Subgroup analysis of single-arm meta-analysis of first test CSTS failure among infants admitted to the A) Nursery and B) NICU

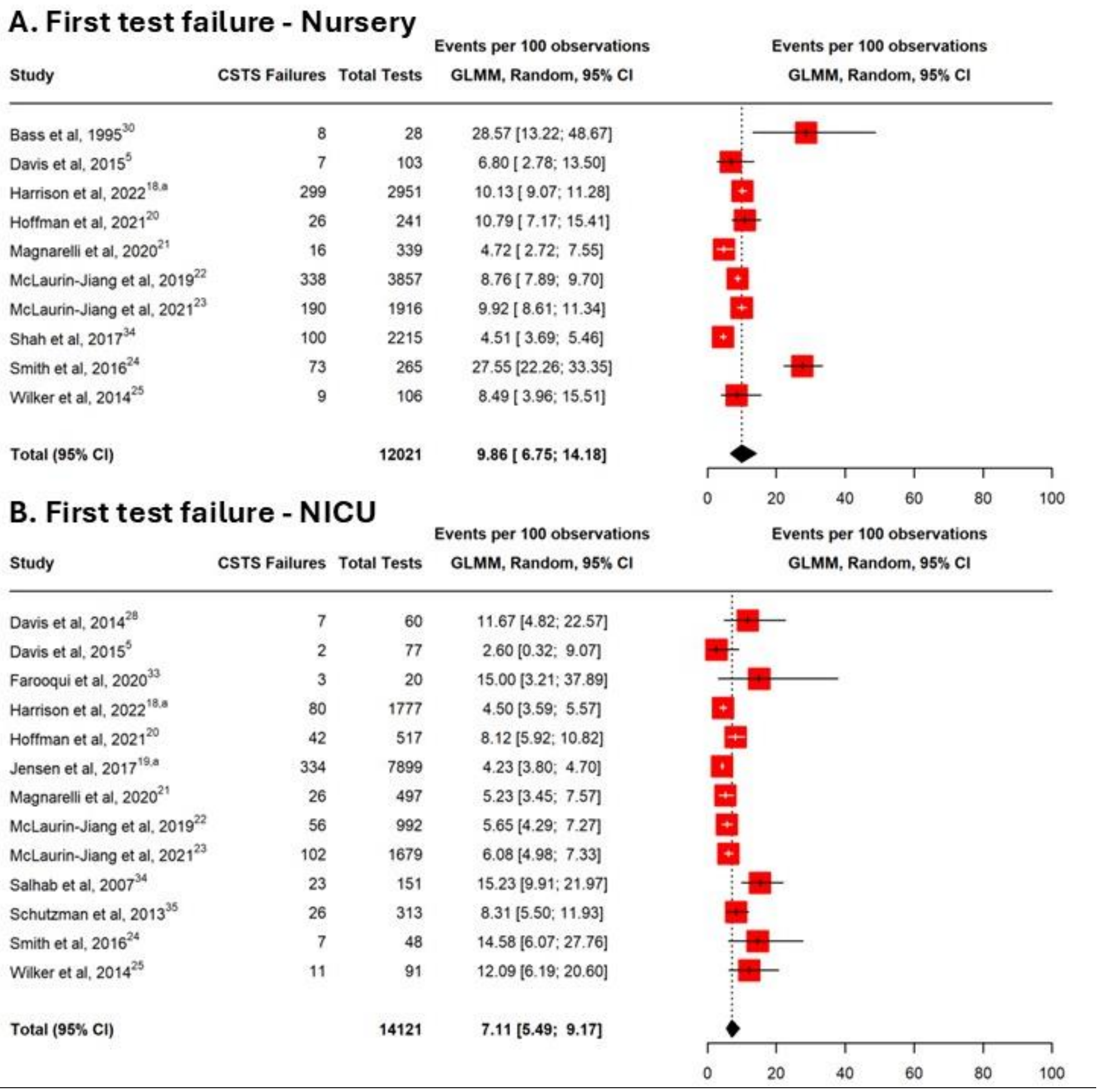

[a]Meta-analysis incorporates CSTS results from patients who underwent CSTS in non-randomized intervention studies which include a comparison group

Abbreviations: CSTS – car seat tolerance screen, GLMM – generalized linear mixed model, CI – confidence interval

© 2026 King BC et al. *JAMA Network Open*.

eFigure 3. Subgroup analysis of single-arm meta-analysis of first test CSTS failure among A) Preterm infants and B) Term infants

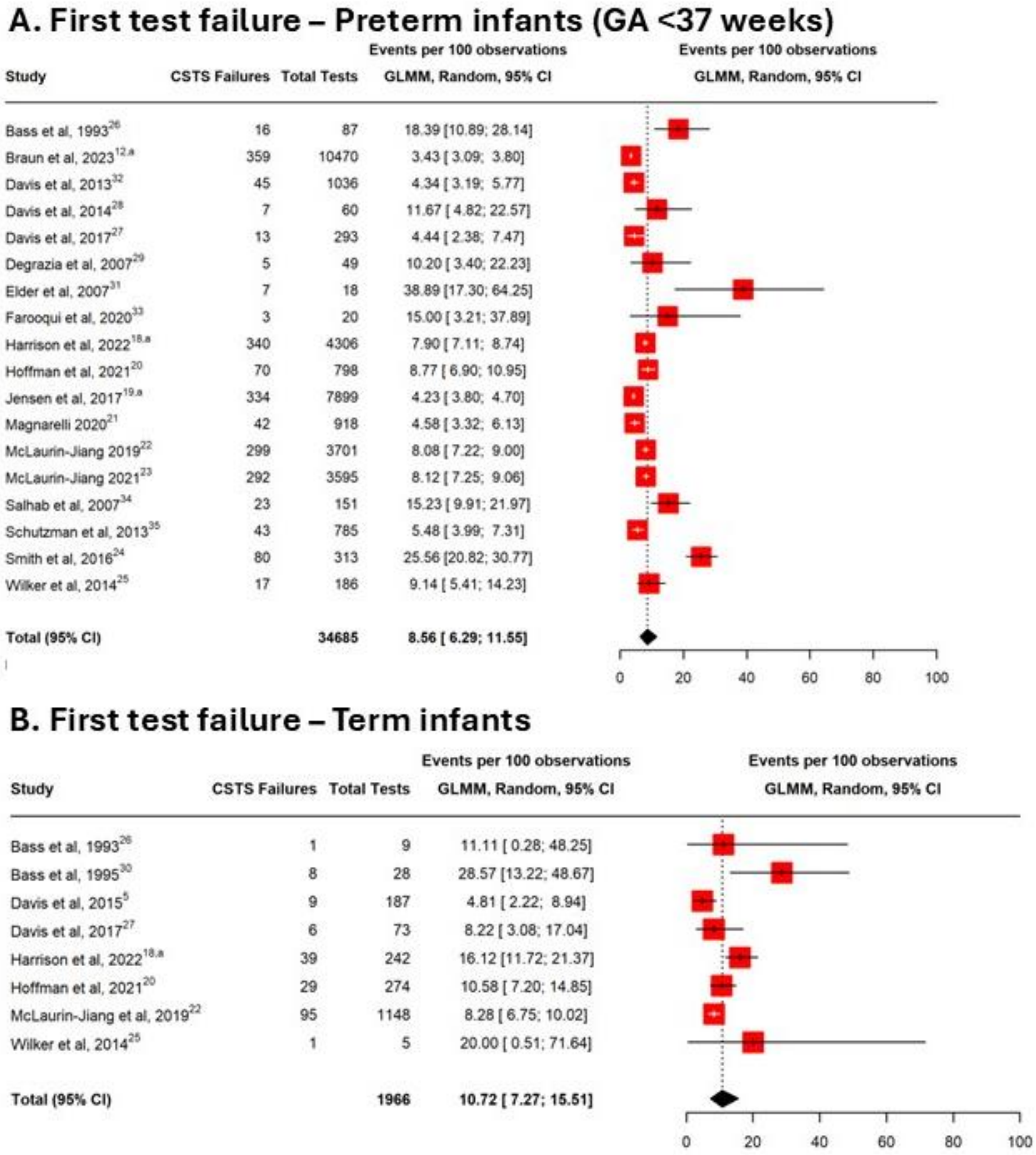

[a]Meta-analysis incorporates CSTS results from patients who underwent CSTS in non-randomized intervention studies which include a comparison group

Abbreviations: CSTS – car seat tolerance screen, GLMM – generalized linear mixed model, CI – confidence interval

© 2026 King BC et al. *JAMA Network Open*.

eFigure 4. Post-discharge outcomes from included studies comparing CSTS vs no CSTS

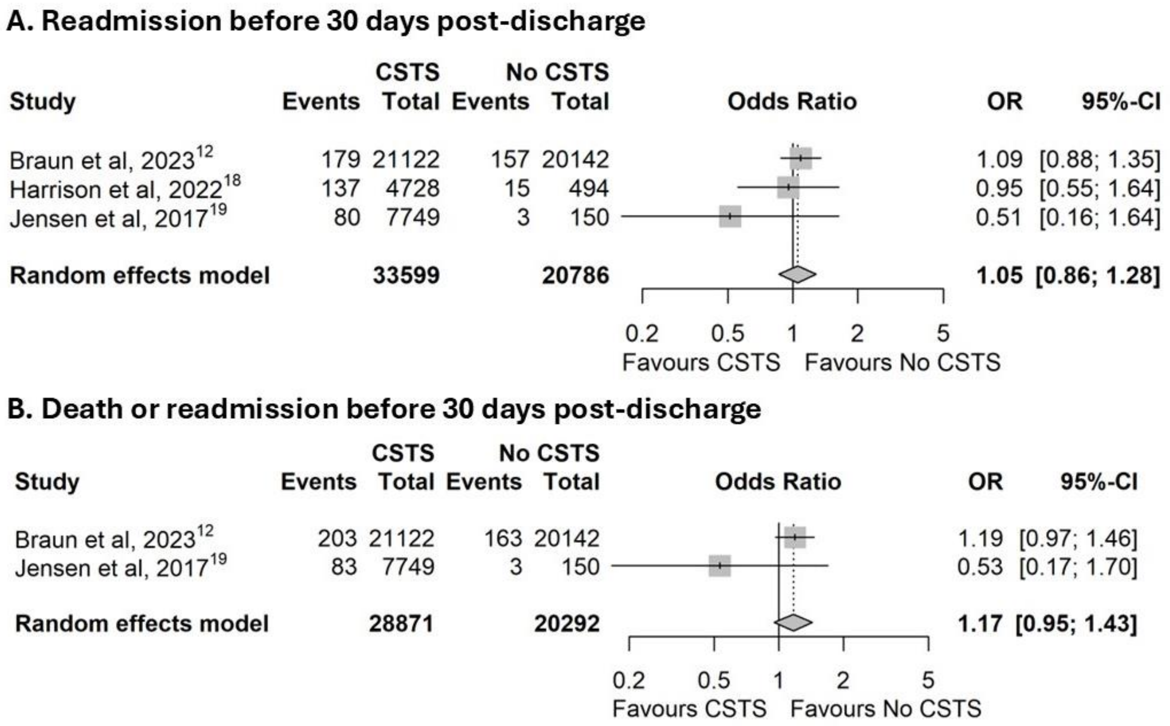

© 2026 King BC et al. *JAMA Network Open*.
