# Supplementary material for: Predischarge Car Seat Tolerance Screening in Preterm and At-Risk Full-Term Infants: A Systematic Review and Meta-Analysis
Source: JAMA Netw Open. 2026 Feb 9;9(2):e2558197. doi: 10.1001/jamanetworkopen.2025.58197 (PMC12887743; doi:10.1001/jamanetworkopen.2025.58197)
Supplement: Supplement 2. — Data Sharing Statement [file jamanetwopen-e2558197-s002.pdf]

Data Sharing Statement

King. Predischarge Car Seat Tolerance Screening in Preterm and At-Risk Full-Term Infants. *JAMA Netw Open*. Published February 09, 2026. doi:10.1001/jamanetworkopen.2025.58197

Data
Data available: Yes
Data types: Data (not involving human participants)
How to access data: Upon request, to bking6@bidmc.harvard.edu
When available: With publication

Supporting Documents
Document types: None

Additional Information
Who can access the data: Anyone requesting data
Types of analyses: Data extraction, forest plots
Mechanisms of data availability: On request
